# Technical Issues of Vim–PSA Double-Target DBS for Essential Tremor

**DOI:** 10.3390/brainsci13040566

**Published:** 2023-03-28

**Authors:** Xusheng Hou, Yixiang Mo, Zhiyuan Zhu, Huan Zhang, Xinzi Liu, Zhihao Zou, Xiaozheng He, Shan Xue, Jiangtao Li, Mengqian Li, Shizhong Zhang

**Affiliations:** 1 Department of Functional Neurosurgery, Zhujiang Hospital, Southern Medical University, Guangzhou 510280, China; 2 Department of Neurosurgery, Affiliated Hospital 2 of Nantong University and First People’s Hospital of Nantong City, Nantong 226001, China; 3 Department of Medical Imaging, Zhujiang Hospital, Southern Medical University, Guangzhou 510280, China; 4 Department of Neurosurgery, General Hospital of Xinjiang Military Command of PLA, Urumqi 830000, China

**Keywords:** essential tremor, deep brain stimulation, ventral intermediate nucleus, posterior subthalamic area, double-target

## Abstract

Background: Deep brain stimulation (DBS) is an effective surgical treatment for essential tremor (ET), with the ventral intermediate nucleus (Vim) and posterior subthalamic area (PSA) as the most common targets. The stimulation efficacy of ET with Vim–PSA double-target DBS has been reported. Herein, we aim to propose surgical techniques for Vim–PSA double-target DBS surgery. Methods: This study enrolled six patients with ET who underwent Vim–PSA double-target electrode implantation from October 2019 to May 2022. The targets were located and adjusted using coordinates and multimodality MRI images. A burr hole was accurately drilled in line with the electrode trajectory under the guidance of a stereotactic frame. Novel approaches were adopted during the electrode implantation process for pneumocephalus reduction, including “arachnoid piamater welding” and “water sealing”. Electrophysiological recording was used to identify the implantation sites of the electrodes. A 3D reconstruction model of electrodes and nuclei was established to facilitate programming. Results: The combination of coordinates and multimodality MRI images for target location and adjustment enabled the alignment of Vim and PSA. Postoperative CT scanning showed that the electrode was precisely implanted. Stereotactic guidance facilitated accurate burr hole drilling. “Arachnoid piamater welding” and “water sealing” were efficient in reducing pneumocephalus. Intraoperative electrophysiological verified the efficacy of Vim–PSA double-target DBS surgery. Conclusions: The methods for target location and adjustment, accurate drilling of the burr hole, reduction in pneumocephalus, and intraoperative electrophysiological verification are key issues in DBS surgery targeting both the Vim and PSA. This study may provide technical support for Vim–PSA DBS, especially for surgeons with less experience in functional neurosurgery.

## 1. Introduction

Essential tremor (ET) is a common movement disorder disease prevalent in those over 65 years of age [1]. Deep brain stimulation (DBS) is an established surgical treatment for ET, especially for patients with severe medically refractory ET. The most well-known DBS targets for ET are the ventral intermediate nucleus (Vim) and the posterior subthalamic area (PSA). The common DBS targets for ET are listed in Table 1.

It remains controversial which target is better in terms of efficacy. The Vim is a traditional DBS surgical target for ET. The short-term (usually less than 1 year) efficacy of Vim-DBS is widely accepted [2,3]. However, several studies have revealed limitations regarding the long-term efficacy of Vim-DBS, including stimulation tolerance and rebound [4,5,6]. The PSA, which includes the prelemniscal radiation (Raprl), caudal zona incerta (cZi), and prerubral field, has become a popular target given its promising therapeutic effects [7,8,9]. Since 2012, several studies have proposed that PSA/cZi is a better target than Vim for tremor alleviation [10,11,12,13]. However, in 2018, two observational studies suggested that Vim-DBS might be more efficient in reducing tremors, and might provide better long-term outcomes than PSA/cZi-DBS [14,15]. Importantly, a randomized double-blind crossover trial performed by Barbe et al. suggested that PSA–DBS is equally as effective as Vim-DBS [16]. This study provides the first instance of Level I evidence for ET. This being the case, the debate regarding which target should be chosen for DBS surgery in patients with ET is still ongoing.

Interestingly, the anatomical locations of the Vim and PSA make it possible to align these two targets with one trajectory. The PSA is anatomically situated ventromedial to the Vim, conforming to the direction (from anterior superior lateral to posterior inferior medial) of the accustomed trajectory. Several institutions have attempted to perform Vim–PSA electrode implantation surgery by aligning the Vim and PSA in a single electrode [17,18,19,20]. These studies suggested that the efficacy of double-target stimulation is similar to that of single-target stimulation (Vim or PSA) in alleviating tremors. In terms of complications and side effects, insignificant differences were found between the double-target and single-target groups. Nevertheless, the studies proposed that double-target DBS has obvious advantages over single-target DBS. Firstly, it would not be necessary to select a target. The possibility of missing the optimal target and potential need for a second surgery would be decreased. Secondly, it enables the intraoperative electrophysiological exploration of both targets, facilitating electrode implantation to the optimal site. Thirdly, multiple contacts would facilitate individualized programming. Patients may obtain better tremor control and have fewer side effects and lessened stimulation tolerance. Finally, future trials can be performed to compare the efficacy of tremor alleviation between the two targets in the same patient.

Although double-target DBS may have multiple advantages, it requires higher precision in electrode implantation surgery. However, specialized descriptions of the surgical techniques for Vim–PSA DBS are lacking. Since 2019, our center has performed Vim–PSA DBS for ET patients, and continues to explore the technical issues of double-target surgery. In practice, we have proposed three major technical challenges in double-target DBS surgery: (1) methods of locating the Vim and PSA and the appropriate adjustment for aligning both targets; (2) methods of ensuring precise electrode implantation into targets along the planned trajectory; (3) verification of the intraoperative electrode implantation site and its corresponding effect. In this study, we explore potential solutions for the above challenges, which we consider to be the key surgical techniques.

This study includes a brief introduction of the relevant background and the major materials and methods, followed by the key results (locating the Vim and PSA targets, target adjustment, accurate burr hole drilling, reducing electrode displacement caused by pneumocephalus and cerebrospinal fluid (CSF) loss, intraoperative microelectrode recording (MER) of neuronal firing patterns, and reconstruction of the electrode and programming strategy) and a discussion. We hope to promote detailed double-target DBS technical support for surgeons.

## 2. Methods and Materials

This section presents the major methods and materials of this study, including participants and study design, image acquisition and pre-processing, target planning, surgical procedure, and data analysis.

### 2.1. Participants and Study Design

We retrospectively reviewed the clinical data of 6 patients with ET who underwent Vim–PSA double-target DBS at our institution from October 2019 to May 2022. This study was conducted with the approval of the Medical Ethics Review Board of Southern Medical University, approval number: 2020-KY-060-01.

### 2.2. Image Acquisition and Pre-Processing

A few days before surgery, a high-resolution magnetic resonance imaging (MRI) scan was performed using a 3 T MRI scanner (Philips Healthcare, Amsterdam, The Netherlands). The MRI sequences for DBS planning included three-dimensional (3D) T1-weighted imaging (T1WI), T2-weighted imaging (T2WI), fast gray matter acquisition T1 inversion recovery (FGATIR), and diffusion tensor imaging (DTI). All images were conducted in the axial direction. Detailed parameters of the preoperative MRI sequences are listed in Table 2 [21,22,23,24]. The dentato-rubro-thalamic tract (DRTT) was delineated using a Fiber Track software package on the Philips IntelliSpace Portal (Version 9.0) platform. On the morning of the surgery, a preoperative computed tomography (CT) scan was conducted with a multislice CT scanner (Brilliance iCT, Philips Healthcare, the Netherlands). The parameters were as follows: FOV = 240 mm, ST = 0.625 mm. During the surgery, an intraoperative CT scan was performed using a portable head CT scanner (CereTom, NeuroLogica, Danvers, MA, USA) after electrodes were fixed. The parameters of the intraoperative CT images were as follows: FOV = 240 mm, ST = 1.25 mm. One week after surgery, a postoperative CT scan was conducted using the Brilliance iCT. The parameters were as follows: FOV = 240 mm, ST = 0.625 mm.

### 2.3. Target Planning

**Figure 1:**
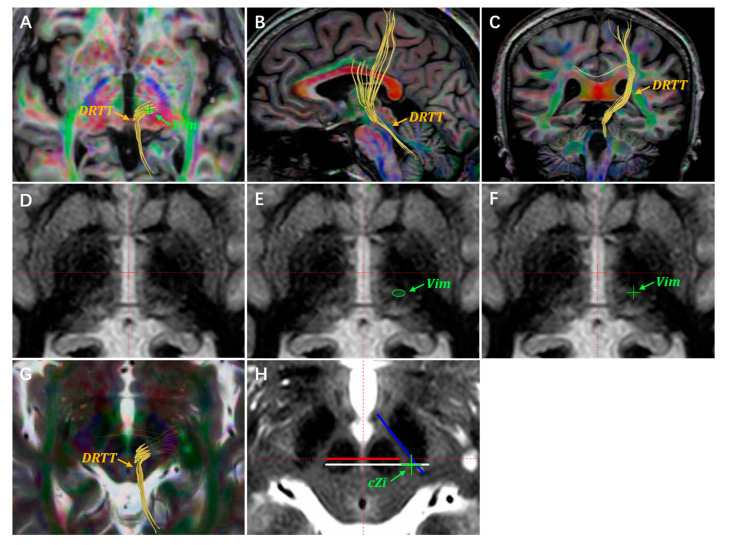
A demonstration of target planning for the Vim and PSA. Targets are located through the atlas-defined stereotactic coordinates, DTI images, and FGATIR images. (**A**) The DRTT is delineated using DTI tractography on the axial anterior commissure–posterior commissure (AC-PC) plane. The Vim target is located on the DTI images, indicated with a green “+”. The DRTT is delineated by the DTI images on the sagittal (**B**) and coronal planes (**C**). (**D**) Unannotated axial FGATIR images, 1 mm below the AC-PC plane. (**E**) The Vim nucleus region is delineated with a green circle on the axial FGATIR images. (**F**) The green “+” represents the planned Vim target. (**G**,**H**) The cZi lies laterally to the DRTT on the most prominent RN layer. The green “+” represents the planned cZi target, which is usually located on the axial T2WI images.

We used the Leksell SurgiPlan system (Elekta, Stockholm, Sweden) to plan the targets and trajectory. The PSA target, which is more ventral, was defined as the primary target. The cZi was selected as the preferred PSA stimulation region. The PSA (cZi) target is located on the most prominent layer of the red nucleus (RN) on the T2WI images [7,8,25]. A horizontal connective line (the red line in Figure 1H) was constructed across the maximum diameter of the bilateral RN. A second line (the blue line in Figure 1H) was drawn along the medial border of the STN. A third line (the white line in Figure 1H) was drawn parallel with the red line passing through the posterior tail of the STN. The PSA (cZi) target was located 1 mm medial to the intersection of the blue and white lines. Regarding the Vim, the stereotactic coordinates were 12–14 mm lateral, 4–6 mm posterior, and 0–1 mm inferior to the MCP [20,23,26]. Both targets were adjusted and verified with the DTI and FGATIR images.

### 2.4. Surgical Procedure

On the morning of the surgery, a Leksell G stereotactic frame (Elekta, Stockholm, Sweden) was attached to the patient’s head under local anesthesia prior to the preoperative CT scan. The Leksell SurgiPlan system was adopted to merge the preoperative CT images and MRI images and to calculate the parameters for electrode implantation. The patient was under local anesthesia during electrode implantation, and was placed in a semi-supine position with the head elevated approximately 15 degrees. Before making the scalp incision, we installed a Leksell stereotactic arc to locate the scalp projective point of the electrode trajectory and designed a forehead curve incision with the projective point as the center. Upon skull exposure, the stereotactic arc was reinstalled to accurately locate a central burr hole point. A standard burr hole with a diameter of 14 mm was drilled with a hand drill. The dura was incised crosswise, with a diameter of approximately 5 mm, leaving the arachnoid intact. After reinstalling the stereotactic arc to locate the cortex entry point, the arachnoid and piamater were coagulated together and incised. During the operation, 0.9% normal saline was continuously dripped into the burr hole at a constant speed (about 2 drops/second), starting from the dura incision until the fixation of the electrode. We utilized intraoperative MER (NeuroTrek system, AlphaOmega, Nof HaGalil, Israel) to trace the neuronal firing patterns of the Vim and PSA. After identifying the border area between the Vim and PSA, a long quadripolar electrode (model 3387, Medtronic, Minneapolis, MN, USA) was implanted at an appropriate site. The patient was awake, and macrostimulation was used to test the efficacy of each contact on the electrode. When the bilateral electrodes were fixed, an intraoperative CT scan was performed to confirm the electrode position and exclude hemorrhage or severe pneumocephalus. After removal of the head frame, an implantable pulse generator (IPG) was implanted into an infraclavicular pocket over the pectoralis fascia under general anesthesia.

### 2.5. Data Analysis

The pneumocephalus region was extracted from the intraoperative CT images, which were printed using 3D Slicer software. The air volume was calculated. The MRI and postoperative CT images were transferred to a computer workstation and reprocessed by the Lead-DBS software toolbox for MATLAB. The 3D reconstruction of electrode(s), Vim, cZi, DRTT, and other related nuclei was performed using the Essential Tremor Probabilistic Mapping Atlas and the Zona Incerta Atlas [27,28].

## 3. Results

This study enrolled six patients with ET who underwent Vim–PSA double-target DBS. The patients included 2 males and 4 females between 54 and 66 years of age. Three patients underwent unilateral electrode implantation, and the others underwent bilateral electrode implantation.

This section presents the major results of this study, including locating the Vim and PSA targets, target adjustment, accurate burr hole drilling, reducing electrode displacement caused by pneumocephalus and CSF loss, intraoperative MER of neuronal firing patterns, and reconstruction of the electrode and programming strategies.

### 3.1. Locating the Vim and PSA Targets

The Vim and PSA are not visible on routine MRI sequences. Anatomically, the PSA is situated medial to the RN and anterolateral to the STN. Since the RN and STN are obviously visible on the T2WI images, the PSA can be targeted indirectly by referring to the RN and STN coordinates. The Vim used to be located using atlas-defined stereotactic coordinates. In a 2019 retrospective study, Morishita et al. proposed that the Vim nucleus could be identified on high-resolution FGATIR images [23]. Furthermore, several studies have suggested that the DTI images could be applied to assist in targeting the Vim [22,29,30]. The DRTT is an interconnecting fiber pathway between the Vim and PSA. By delineating and visualizing the DRTT, it would be feasible to target the Vim. In addition, the DRTT has been proposed as an anatomical structure for stimulation-induced tremor alleviation [22,30,31]. Advanced MRI sequences such as quantitative susceptibility mapping (QSM) may have the potential to visualize the Vim nucleus, but determining the reliability and accuracy of this method will require further testing [24,32]. Our center currently locates the Vim and PSA through the stereotactic coordinates, FGATIR imaging, and DTI tractography. In Figure 1, we depict our experience in targeting the Vim and PSA with data from one of our patients. This is a 71-year-old patient with ET who underwent unilateral Vim–PSA double-target DBS surgery in 2020 (Figure 1).

### 3.2. Targets Adjustment

Upon obtaining patient consent, we implanted double-target electrodes in patients with ET. Making subtle adjustments for double-target analysis is challenging, but it is often necessary during the operation. When the widths of the lateral ventricle and third ventricle are enlarged (such as due to brain atrophy in the elderly), the Vim target correspondingly shifts laterally. This may increase the coronal angle of the double-target trajectory. If the coronal angle exceeds 30 degrees, the risk of the trajectory traversing critical brain regions greatly increases. This includes the frontal language area, internal capsule, and other functional areas. When the locations of both targets have been confirmed, the coronal and sagittal angles of the double-target trajectory are fixed. The only way to adjust the trajectory is by making subtle adjustments to the locations of both targets. However, there is currently a lack of specialized descriptions for target adjustment. Papavassiliou et al. proposed that the optimal electrode location for Vim-DBS corresponds to the anterior margin of the Vim [33]. The authors noted that electrode deviations more than 2 mm from the optimal coordinate were associated with poor tremor control. Cury et al. agreed with this assessment and proposed that stimulations of the Vim caudal area were associated with worse results regarding tremor control. Regarding the PSA, stimulation of the cZi and Raprl has been proven to be equally effective in tremor control [7,9,22]. Based on our experience, we have proposed two basic principles for the subtle adjustment of the two targets, as follows.

Firstly, when the sagittal angle of the trajectory is large, so that the electrode may traverse the motor area or other functional areas, the Vim target may be adjusted in the anterior direction within a 1 mm range.

**Figure 2:**
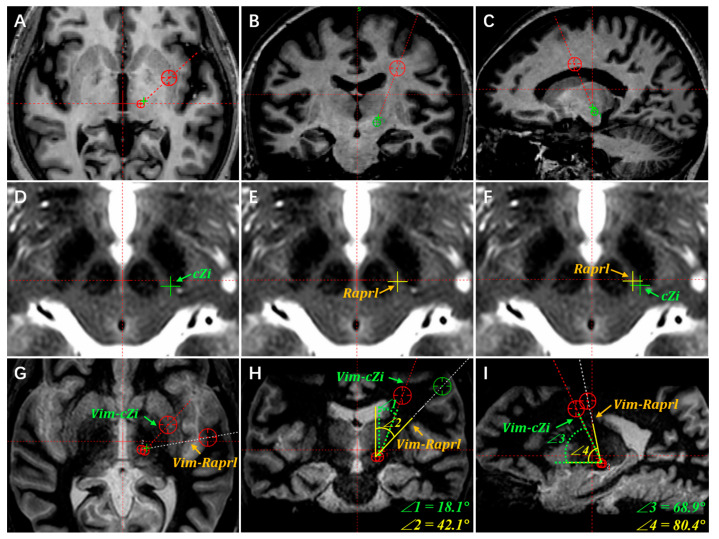
The cZi is selected as the preferred PSA region for alignment of the Vim and PSA. (**A**) The default coronal and sagittal angles of trajectory are usually preset on the axial plane. The coronal and sagittal angles are adjusted to ensure that the trajectory traverses the target of Vim on the coronal (**B**) and sagittal planes (**C**). (**D**) The cZi target is marked with a green “+” on the axial T2WI images. (**E**) The common stimulated area of Raprl for ET is marked with a yellow “+” on the T2WI images. (**F**) The Raprl target lies medial and anterior to the cZi on the same plane of the axial T2WI images. To illustrate the selection of the stimulation region in the PSA, the planned Vim–cZi and Vim–Raprl trajectories are compared on the axial (**G**), coronal (**H**), and sagittal (**I**) planes, respectively. In this case, the Vim stereotactic coordinates are 13.7 mm lateral, 5.6 mm posterior, and 1.0 mm inferior relative to the MCP. The cZi stereotactic coordinates are 12.5 mm lateral, 6.9 mm posterior, and 4.0 mm inferior relative to the MCP. As for the Raprl, the stereotactic coordinates are 10.6 mm lateral, 6.1 mm posterior, and 4.0 mm inferior relative to the MCP. Correspondingly, the coronal and sagittal angles of the Vim–cZi trajectory are, respectively, 18.1 degrees and 68.9 degrees (∠1 and ∠3 in Figure 2H,I). The coronal and sagittal angles of the Vim–Raprl trajectory are, respectively, 42.1 degrees and 80.4 degrees (∠2 and ∠4 in Figure 2H,I). Both the coronal and sagittal angles of the Vim–Raprl trajectory are significantly larger than those of the Vim–cZi trajectory. Meanwhile, the Vim–Raprl trajectory traverses the internal capsule and insular cortex.

Secondly, the cZi is generally selected as the preferred PSA stimulation region. Since the cZi anatomically lies lateral to the Raprl, the trajectory of the Vim–cZi double target has a smaller coronal angle than that of the Vim–Raprl. This anatomical location decreases the potential of the electrode traversing the insular cortex, internal capsule, and language area. Figure 2 illustrates the alignment and adjustment procedure for Vim–PSA double-target surgery based on information from the same patient in Figure 1.

### 3.3. Accurate Burr Hole Drilling

Upon completing the Vim–PSA double-target planning, the burr hole location and cerebral cortex entry point were confirmed. The accurate location of the burr hole was a prerequisite for the accurate alignment of both targets along the planned trajectory. Even a subtle deviation may lead to failure to align both targets. A curvilinear scalp incision on the forehead was designed after the projective trajectory point was verified using the stereotactic arc. This was carried out to minimize the incision and avoid a scalp split due to high tension. To accurately drill the burr hole, the burr hole location was confirmed with the guidance of the stereotactic arc. The incision sites of the dura and cerebral cortex were located using the same method. As CSF loss is a concern in terms of the dura and cortex incisions, the process is described in the following section.

### 3.4. Reducing Electrode Displacement Caused by Pneumocephalus and CSF Loss

Pneumocephalus and CSF loss are critical causes of brain shift and electrode displacement [34,35]. Different strategies have been adopted to overcome the hazards of pneumocephalus and CSF loss, mainly including reducing intracranial pressure and sealing the burr hole or dura [36,37,38]. Patients are commonly placed in a semi-supine position to lower intracranial pressure. The arachnoid incision is a direct cause of pneumocephalus and CSF loss. We have promoted a distinctive technique for the arachnoid incision, named “arachnoid piamater welding”. In this study, the dura was incised crossly and coagulated with a small hole approximately 5 mm in diameter, leaving the arachnoid intact. Hence, the size of the remaining dura was able to act as a loading plane for fibrin glue. The intact arachnoid was welded and attached to the piamater. Then, the piamater was coagulated and incised with the cortex to avoid an excessive downward shift of the cortex during brain penetration. Since the subarachnoid space was intact during the operation, this could be a possible method for reducing CSF loss.

**Figure 3:**
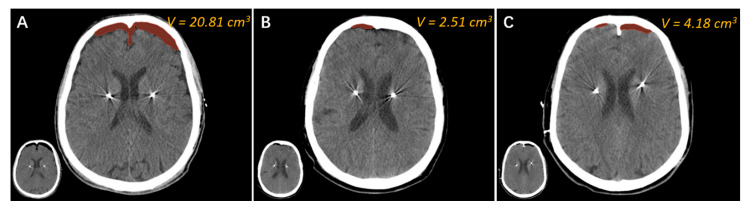
The comparison of three methods to reduce pneumocephalus and CSF loss. Representative intraoperative CT images of three patients who underwent bilateral electrode implantation, each with one of three burr hole-sealing approaches, are presented. (**A**) A 58-year-old male patient with Parkinson’s disease (PD) underwent bilateral STN-DBS electrode implantation in 2013. During the operation, bone wax was used to seal the burr hole. The air volume of the intraoperative CT images was 20.81 cm^3^. (**B**) A 65-year-old female patient with ET underwent bilateral Vim-DBS electrode implantation in 2021. The burr hole was filled with fibrin glue during the surgery. The air volume of the intraoperative CT images was 2.51 cm^3^. (**C**) A 60-year-old female patient with ET underwent bilateral Vim–PSA DBS electrode implantation in 2022. We adopted the “water sealing” method for this patient. The air volume of the intraoperative CT images was 4.18 cm^3^.

Moreover, various materials have been utilized to seal the burr hole, mainly including bone wax, gel foam, and fibrin glue. Fibrin glue sealing has been widely approved as an effective method for reducing pneumocephalus and CSF loss [36,37,38]. Our observations concur with this assessment, as we also observed a satisfactory sealing effect with fibrin glue (Figure 3B). However, it may induce allergic reactions or spread viruses, and is of poor economic benefit [39,40].

We recently adopted a novel approach called “water sealing” to reduce pneumocephalus and CSF loss in DBS surgery. During the operation, 0.9% normal saline was continuously dripped into the burr hole at a constant speed (about 2 drops/second), starting from the dura incision until the fixation of the electrode. Herein, we present three cases using the methods of bone wax sealing, fibrin glue sealing, and “water sealing” (Figure 3). It can be seen that the air volume in the patient who underwent “bone wax sealing” was significantly greater than that of the other two, suggesting that bone wax is unsatisfactory for burr hole sealing (Figure 3A). The air volume of the patient who underwent “fibrin glue sealing” appeared to be the lowest among the three patients (Figure 3B). Finally, the air volume of the patient who underwent “water sealing” was approximately the same as that achieved by “fibrin glue sealing” and obviously less than that achieved by “bone wax sealing” (Figure 3C). We surmise that the “water sealing” technique is comparable with expensive “fibrin glue sealing”. Given the superior economic impact of “water sealing”, we recommend using this novel burr hole sealing method for DBS as well as other neurosurgeries.

### 3.5. Intraoperative MER of Neuronal Firing Patterns

**Figure 4:**
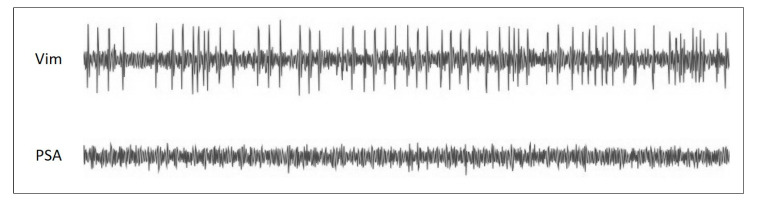
The canonical neuronal firing patterns of the Vim and PSA. The neuronal firing patterns from intraoperative MER are among the critical factors for selecting the electrode implantation site for the Vim–PSA double-target DBS surgery. Generally, the high background firing activity of neurons corresponding to tremors can be recorded in the Vim nucleus. On the other hand, the neuronal firing pattern of the PSA is characterized by low background noise and sparse neuronal firing.

It is well accepted that the MER can enhance the accuracy of targeting the deep brain nucleus. Given that general anesthesia may compromise the signals of certain nuclei, we suggest local anesthesia for Vim–PSA DBS surgery. The intraoperative MER can be applied to record the neuronal firing patterns of different sites, distinguishing the borders of the Vim and PSA. A typical neuronal pattern of the Vim nucleus is high background firing activity with large amplitude spikes corresponding to tremors (Figure 4). In contrast, relatively low neuronal firing activity with a silent background is recorded in the PSA. After identifying the border area between the Vim and PSA, an appropriate site is selected at which to implant the electrode. To ensure the coverage of both targets, a long electrode should be chosen. Macrostimulation is utilized to test the efficacy of each contact of the electrode. By using local anesthesia, the surgeons are able not only to directly confirm the effects of the electrode implantation, but also to obtain a reference for postoperative programming.

### 3.6. Reconstruction of Electrode and Programming Strategy

**Figure 5:**
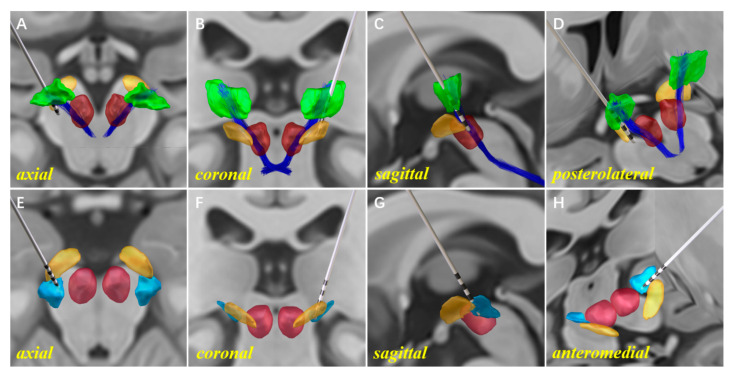
An illustration of the 3D reconstruction models for unilateral Vim–PSA double-target DBS. A single electrode was implanted into the Vim–PSA double target of the left hemisphere. The 3D reconstruction of the electrode, the Vim (green), the cZi (light blue), the STN (orange), the RN (red), and the DRTT (dark blue) are shown on the axial (**A**,**E**), coronal (**B**,**F**), and sagittal (**C**,**G**) sections. For a clearer illustration of the relative positions of the electrode implanted into the Vim and PSA, perspectives are displayed from the posterolateral (**D**) and anteromedial (**H**) directions.

The use of Lead-DBS software for 3D reconstruction helps us to progress from planar medical images to stereo models of the electrode and related nuclei. This method reveals the relative anatomical relationship between the nucleus and each electrode contact, which facilitates postoperative programming (Figure 5). In this study, analysis of the relative location between the electrode contacts and nuclei was performed prior to the formulation of the programming strategy. Information from the ET patient mentioned in Figure 1 was used here. In the 3D reconstruction models, the upper two contacts of the electrode were implanted into the anterior region of the Vim, with proximity to the DRTT and matching the surgery plan (Figure 5A–D). It was observed that only three-quarters of the lowest electrode contact were implanted into the cZi (Figure 5E–H). We propose two possible explanations for this situation. Firstly, the contact length of the model 3387 electrode was insufficient to cover the Vim–PSA double target. A longer electrode with more contacts might facilitate the double-target surgery. Secondly, although the intraoperative MER could distinguish the traversing site from the Vim to the PSA, it had certain difficulties in determining the implantation depth of the electrode due to the silent background firing pattern of the PSA. Stimulation tests were conducted to explore the efficacy and side effects of each contact one month after the surgery. The stimulation parameters with good outcomes were as follows: (Vim) 10–C+, voltage = 2.1 V, pulse width = 60 µs, frequency = 135 Hz; (PSA) 8–C+, voltage = 1.7 V, pulse width = 90 µs, frequency = 160 Hz. No significant side effects were observed. The stimulation voltage of the Vim was higher than that of the PSA. However, given that the Vim might have better short-term efficacy, we developed a strategy with the Vim as the primary stimulation region in the early stage.

## 4. Discussion

This paper aims to explore the technical issues surrounding Vim–PSA double-target DBS for ET treatment. It was inspired by reports from institutions implanting DBS electrodes for Vim–PSA double-targeting as an ET treatment. With DBS regarded as a promising therapy for ET, we are greatly interested in the novel approach of Vim–PSA double-target DBS. Reports of double-target surgery are not common, and few technical issues have been described in detail. In this study, we thoroughly describe the technical challenges of double-target surgery, intending to raise awareness of the corresponding technical issues for surgeons.

### 4.1. Advantages

The implantation of one electrode to align the two most popular DBS targets for ET treatment is an ingenious and promising development. Some research has argued for directly locating targets via DTI tractography [22,26,41]. The high resolution FGATIR sequence has been reported as being capable of identifying the Vim nucleus. However, it should be stated that the fibers delineated on the DTI images are arbitrary and numerous, according to the selection of region of interest (ROI). Moreover, we found it unsatisfactory to depict the border of the Vim using only the FGATIR images, which often needed to be associated with the DTI images. This being the case, there may be a certain risk in conducting direct targeting by images at present. Herein, we propose that Vim–PSA double-target surgery be pre-planned through routine MRI images. Then, the targets and trajectory can be adjusted and verified using the FGATIR and DTI images.

The accuracy of electrode implantation is intimately associated with clinical efficacy. Unlike single target DBS surgery, the trajectory of the electrode for double-target DBS is determined while the target planning is accomplished. Aside from accurately drilling the burr hole and locating the cortex entry point, it is critical to minimize various interference factors that would cause electrode displacement. With the current immature surgical technique, it is essential to verify the implantation site of the electrode through various methods. The intraoperative MER, under local anesthesia, is capable of distinguishing the border of the nucleus and fiber tract, thus facilitating the selection of the electrode implantation site. The awake macrostimulation test can then enhance the reliability of the selection. Intraoperative images, including MRI and CT, are utilized by some institutions to verify the depth of the electrode implantation. The intraoperative MRI is usually obtained using a 1.5 T MRI scanner rather than a 3 T MRI scanner. This lessens the risk of brain damage and electrode displacement. However, the low-field-strength MRI provides poor resolution images, making it difficult to distinguish the nucleus and the fiber tract. Since the intraoperative CT images are of low soft tissue contrast, they are commonly fused with the preoperative MRI images to discern the electrode implantation site into the nucleus. Due to the brain shift caused by pneumocephalus and CSF loss, there is a certain deviation between the nucleus and electrode on the MRI–CT fusion images. We suggest that the postoperative CT images obtained one week after surgery be fused with the preoperative MRI images. Given the disappearance of pneumocephalus on the postoperative CT images, these fusion images reliably demonstrates the position relationship between the electrode and nucleus. Furthermore, reprocessing the postoperative CT images and preoperative MRI images using Lead-DBS software can facilitate postoperative programming. The anatomic relationship between each contact and nucleus, as obtained through the 3D reconstruction models, would be straight. 

### 4.2. Limitations

The number of recruited cases for this study is relatively insufficient. At present, we have located the Vim and PSA via target coordinates and multimodality MRI. The FGATIR or DTI single MRI sequence is not able to locate targets accurately. However, the advanced QSM MRI sequence is reported as being able to clearly visualize the Vim. This inspires further improvements to be made to the preoperative MRI protocol for surgery. We developed novel approaches for arachnoid incision and burr hole sealing with the aim of reducing pneumocephalus and CSF loss. The efficacy of these novel approaches has only been demonstrated through limited surgical cases. A comparative study on the efficacy of novel approaches and accustomed approaches should be performed in the future.

## 5. Conclusions

Vim–PSA double-target DBS is a promising therapeutic method for ET. The technical issues include target locating and adjustment, accurate burr hole drilling, pneumocephalus reduction, and intraoperative electrophysiological verification. These points may provide technical support for Vim–PSA DBS, especially for surgeons with less experience in this area.

## Figures and Tables

**Table 1:** DBS targets for ET.

	Location or Component	Coordinates (Relative to the Midcommissural Point (MCP))
Vim	Thalamus	12~14 mm lateral 4~6 mm posterior 0~1 mm inferior
PSA	Caudal zona incerta, prelemniscal radiation, prerubral field	10~12 mm lateral 6~8 mm posterior 2~4 mm inferior
Globus pallidus interna (GPi)	Medial to the globus pallidus external (GPe)	19~21 mm lateral 2~4 mm anterior 4~6 mm inferior
Subthalamic nucleus (STN)	Superior to the substantia nigra (SN)	10~12 mm lateral 2~4 mm posterior 3~4 mm inferior

**Table 2:** Parameters of preoperative MRI.

3D T1WI	Repetition time (TR) = 7.9 ms Echo time (TE) = 3.8 ms Field of view (FOV) = 256 mm Matrix = 256 × 256 Slice thickness (ST) = 1 mm, 160 slices
T2WI	TR = 4070 ms, TE = 100 ms FOV = 240 mm, Matrix = 256 × 256 ST = 2 mm, 80 slices
FGATIR	TR = 3000 ms, TE = 4.39 ms FOV = 240 mm, Matrix = 240 × 240 ST = 1.0 mm, 120 slices Bandwidth = 140 Hz/pixel
DTI	Gradient directions = 33 TR = 7500 ms, TE = 84 ms FOV = 240 mm, Matrix = 128 × 128 ST = 2 mm, 70 slices B value = 1000 s/mm^2^

## Data Availability

Not applicable.

## References

[1] Shanker V. (2019). Essential tremor: Diagnosis and management. BMJ.

[2] Dallapiazza R.F., Lee D.J., De Vloo P., Fomenko A., Hamani C., Hodaie M., Kalia S.K., Fasano A., Lozano A.M. (2019). Outcomes from stereotactic surgery for essential tremor. J. Neurol. Neurosurg. Psychiatry.

[3] Cury R.G., Fraix V., Castrioto A., Perez F.M., Krack P., Chabardes S., Seigneuret E., Alho E., Benabid A.L., Moro E. (2017). Thalamic deep brain stimulation for tremor in Parkinson disease, essential tremor, and dystonia. Neurology.

[4] Barbe M.T., Liebhart L., Runge M., Pauls K.A., Wojtecki L., Schnitzler A., Allert N., Fink G.R., Sturm V., Maarouf M. (2011). Deep brain stimulation in the nucleus ventralis intermedius in patients with essential tremor: Habituation of tremor suppression. J. Neurol..

[5] Shih L.C., LaFaver K., Lim C., Papavassiliou E., Tarsy D. (2013). Loss of benefit in VIM thalamic deep brain stimulation (DBS) for essential tremor (ET): How prevalent is it?. Parkinsonism Relat. Disord..

[6] Paschen S., Forstenpointner J., Becktepe J., Heinzel S., Hellriegel H., Witt K., Helmers A.K., Deuschl G. (2019). Long-term efficacy of deep brain stimulation for essential tremor: An observer-blinded study. Neurology.

[7] Blomstedt P., Sandvik U., Tisch S. (2010). Deep brain stimulation in the posterior subthalamic area in the treatment of essential tremor. Mov. Disord..

[8] Plaha P., Khan S., Gill S.S. (2008). Bilateral stimulation of the caudal zona incerta nucleus for tremor control. J. Neurol. Neurosurg. Psychiatry.

[9] Nowacki A., Debove I., Rossi F., Schlaeppi J.A., Petermann K., Wiest R., Schupbach M., Pollo C. (2018). Targeting the posterior subthalamic area for essential tremor: Proposal for MRI-based anatomical landmarks. J. Neurosurg..

[10] Sandvik U., Koskinen L.O., Lundquist A., Blomstedt P. (2012). Thalamic and subthalamic deep brain stimulation for essential tremor: Where is the optimal target?. Neurosurgery.

[11] Holslag J., Neef N., Beudel M., Drost G., Oterdoom D., Kremer N.I., van Laar T., van Dijk J. (2018). Deep Brain Stimulation for Essential Tremor: A Comparison of Targets. World Neurosurg..

[12] Fan H., Bai Y., Yin Z., An Q., Xu Y., Gao Y., Meng F., Zhang J. (2022). Which one is the superior target? A comparison and pooled analysis between posterior subthalamic area and ventral intermediate nucleus deep brain stimulation for essential tremor. CNS Neurosci. Ther..

[13] Kvernmo N., Konglund A.E., Reich M.M., Roothans J., Pripp A.H., Dietrichs E., Volkmann J., Skogseid I.M. (2022). Deep Brain Stimulation for Arm Tremor: A Randomized Trial Comparing Two Targets. Ann. Neurol..

[14] Eisinger R.S., Wong J., Almeida L., Ramirez-Zamora A., Cagle J.N., Giugni J.C., Ahmed B., Bona A.R., Monari E., Wagle S.A. (2018). Ventral Intermediate Nucleus Versus Zona Incerta Region Deep Brain Stimulation in Essential Tremor. Mov. Disord. Clin. Pract..

[15] Degeneffe A., Kuijf M.L., Ackermans L., Temel Y., Kubben P.L. (2018). Comparing deep brain stimulation in the ventral intermediate nucleus versus the posterior subthalamic area in essential tremor patients. Surg. Neurol. Int..

[16] Barbe M.T., Reker P., Hamacher S., Franklin J., Kraus D., Dembek T.A., Becker J., Steffen J.K., Allert N., Wirths J. (2018). DBS of the PSA and the VIM in essential tremor. Neurology.

[17] Chang W.S., Chung J.C., Kim J.P., Chang J.W. (2013). Simultaneous Thalamic and Posterior Subthalamic Electrode Insertion With Single Deep Brain Stimulation Electrode for Essential Tremor. Neuromodulation Technol. Neural Interface.

[18] Dos S.G.M., Ibarra M., Alho E., Reis P.R., Lopez C.W., Hamani C., Fonoff E.T. (2018). Double-target DBS for essential tremor: 8-contact lead for cZI and Vim aligned in the same trajectory. Neurology.

[19] Bot M., van Rootselaar F., Contarino M.F., Odekerken V., Dijk J., de Bie R., Schuurman R., van den Munckhof P. (2018). Deep Brain Stimulation for Essential Tremor: Aligning Thalamic and Posterior Subthalamic Targets in 1 Surgical Trajectory. Oper. Neurosurg..

[20] Diaz A., Cajigas I., Cordeiro J.G., Mahavadi A., Sur S., Di Luca D.G., Shpiner D.S., Luca C.C., Jagid J.R. (2020). Individualized Anatomy-Based Targeting for VIM-cZI DBS in Essential Tremor. World Neurosurg..

[21] Coenen V.A., Allert N., Mädler B. (2011). A role of diffusion tensor imaging fiber tracking in deep brain stimulation surgery: DBS of the dentato-rubro-thalamic tract (drt) for the treatment of therapy-refractory tremor. Acta Neurochir..

[22] Coenen V.A., Allert N., Paus S., Kronenburger M., Urbach H., Madler B. (2014). Modulation of the cerebello-thalamo-cortical network in thalamic deep brain stimulation for tremor: A diffusion tensor imaging study. Neurosurgery.

[23] Morishita T., Higuchi M., Kobayashi H., Abe H., Higashi T., Inoue T. (2019). A retrospective evaluation of thalamic targeting for tremor deep brain stimulation using high-resolution anatomical imaging with supplementary fiber tractography. J. Neurol. Sci..

[24] Shah B.R., Lehman V.T., Kaufmann T.J., Blezek D., Waugh J., Imphean D., Yu F.F., Patel T.R., Chitnis S., Dewey R.B. (2020). Advanced MRI techniques for transcranial high intensity focused ultrasound targeting. Brain.

[25] Blomstedt P., Stenmark P.R., Hariz G.M., Linder J., Fredricks A., Haggstrom B., Philipsson J., Forsgren L., Hariz M. (2018). Deep brain stimulation in the caudal zona incerta versus best medical treatment in patients with Parkinson’s disease: A randomised blinded evaluation. J. Neurol. Neurosurg. Psychiatry.

[26] Fenoy A.J., Schiess M.C. (2017). Deep Brain Stimulation of the Dentato-Rubro-Thalamic Tract: Outcomes of Direct Targeting for Tremor. Neuromodulation Technol. Neural Interface.

[27] Lau J.C., Xiao Y., Haast R.A.M., Gilmore G., Uludağ K., MacDougall K.W., Menon R.S., Parrent A.G., Peters T.M., Khan A.R. (2020). Direct visualization and characterization of the human zona incerta and surrounding structures. Hum. Brain Mapp..

[28] Nowacki A., Barlatey S., Al-Fatly B., Dembek T., Bot M., Green A.L., Kübler D., Lachenmayer M.L., Debove I., Segura-Amil A. (2022). Probabilistic Mapping Reveals Optimal Stimulation Site in Essential Tremor. Ann. Neurol..

[29] Sedrak M., Gorgulho A., Frew A., Behnke E., DeSalles A., Pouratian N. (2011). Diffusion tensor imaging and colored fractional anisotropy mapping of the ventralis intermedius nucleus of the thalamus. Neurosurgery.

[30] Chazen J.L., Sarva H., Stieg P.E., Min R.J., Ballon D.J., Pryor K.O., Riegelhaupt P.M., Kaplitt M.G. (2018). Clinical improvement associated with targeted interruption of the cerebellothalamic tract following MR-guided focused ultrasound for essential tremor. J. Neurosurg..

[31] Lehman V.T., Lee K.H., Klassen B.T., Blezek D.J., Goyal A., Shah B.R., Gorny K.R., Huston J., Kaufmann T.J. (2020). MRI and tractography techniques to localize the ventral intermediate nucleus and dentatorubrothalamic tract for deep brain stimulation and MR-guided focused ultrasound: A narrative review and update. Neurosurg. Focus..

[32] Deistung A., Schäfer A., Schweser F., Biedermann U., Turner R., Reichenbach J.R. (2013). Toward in vivo histology: A comparison of quantitative susceptibility mapping (QSM) with magnitude-, phase-, and R2⁎-imaging at ultra-high magnetic field strength. Neuroimage.

[33] Papavassiliou E., Rau G., Heath S., Abosch A., Barbaro N.M., Larson P.S., Lamborn K., Starr P.A. (2004). Thalamic deep brain stimulation for essential tremor: Relation of lead location to outcome. Neurosurgery.

[34] Morishita T., Hilliard J.D., Okun M.S., Neal D., Nestor K.A., Peace D., Hozouri A.A., Davidson M.R., Bova F.J., Sporrer J.M. (2017). Postoperative lead migration in deep brain stimulation surgery: Incidence, risk factors, and clinical impact. PLoS ONE.

[35] Sillay K.A., Kumbier L.M., Ross C., Brady M., Alexander A., Gupta A., Adluru N., Miranpuri G.S., Williams J.C. (2013). Perioperative Brain Shift and Deep Brain Stimulating Electrode Deformation Analysis: Implications for rigid and non-rigid devices. Ann. Biomed. Eng..

[36] Takumi I., Mishina M., Hironaka K., Oyama K., Yamada A., Adachi K., Hamamoto M., Kitamura S., Yoshida D., Teramoto A. (2013). Simple solution for preventing cerebrospinal fluid loss and brain shift during multitrack deep brain stimulation surgery in the semisupine position: Polyethylene glycol hydrogel dural sealant capping: Rapid communication. Neurol. Med. Chir..

[37] Sasaki T., Agari T., Kuwahara K., Kin I., Okazaki M., Sasada S., Shinko A., Kameda M., Yasuhara T., Date I. (2018). Efficacy of Dural Sealant System for Preventing Brain Shift and Improving Accuracy in Deep Brain Stimulation Surgery. Neurol. Med-Chir..

[38] Piacentino M., Beggio G., Rustemi O., Zambon G., Pilleri M., Raneri F. (2021). Pneumocephalus in subthalamic deep brain stimulation for Parkinson’s disease: A comparison of two different surgical techniques considering factors conditioning brain shift and target precision. Acta Neurochir..

[39] Albala D.M. (2003). Fibrin sealants in clinical practice. Cardiovasc. Surg..

[40] Beudert M., Gutmann M., Luhmann T., Meinel L. (2022). Fibrin Sealants: Challenges and Solutions. Acs Biomater. Sci. Eng..

[41] Schlaier J., Anthofer J., Steib K., Fellner C., Rothenfusser E., Brawanski A., Lange M. (2015). Deep Brain Stimulation for Essential Tremor: Targeting the Dentato-Rubro-Thalamic Tract?. Neuromodulation Technol. Neural Interface.

